# Quantifying segmentation sensitivity in OCTA: Device-specific profiles across three commercial platforms

**DOI:** 10.1371/journal.pone.0343605

**Published:** 2026-02-27

**Authors:** Michael Hafner, Bettina von Livonius, Daniel Deschler, Siegfried G. Priglinger, Maximilian J. Gerhardt

**Affiliations:** Department of Ophthalmology, LMU University Hospital, Ludwig-Maximilians-Universität München, Munich, Germany; Transilvania University of Brasov: Universitatea Transilvania din Brasov, ROMANIA

## Abstract

Optical coherence tomography angiography (OCTA) enables quantitative assessment of the retinal microvasculature structure, but measurements depend on accurate layer segmentation. We quantified how sensitive commonly used OCTA metrics are to small, systematic shifts of the boundary separating the superficial and deep capillary plexus slabs, and assessed whether the resulting changes are clinically meaningful. In this prospective cross-sectional study, 32 eyes from 16 healthy participants underwent 3 × 3 mm OCTA imaging on three commercial devices (Intalight DREAM, Zeiss Cirrus, Topcon Triton). Using each device’s native segmentation tools, the shared inner plexiform layer/inner nuclear layer boundary was shifted generating five offset conditions (−10, −5, 0, + 5, and +10 μm); 0 μm represents the unshifted standardized definition. En face images were analyzed with the open-source OCTAVA pipeline to derive vessel area density, total vessel length, vessel length density, branching measures, and foveal avascular zone (FAZ) area in both plexuses. Linear mixed-effects models estimated the effect of offset on each metric, expressed as standardized slopes and relative change per 10 μm. Sensitivity to segmentation offset was strongly device- and layer-dependent. DREAM showed pronounced offset effects in both plexuses (e.g., superficial vessel area density +4.9% per 10 μm and deep vessel area density −5.2% per 10 μm), with marked deep FAZ enlargement (+15.5% per 10 μm). Cirrus exhibited moderate deep-plexus sensitivity (approximately −2% per 10 μm for vessel density/length metrics) with high FAZ sensitivity (+16.1% per 10 μm). Triton metrics were comparatively stable, except for deep FAZ area (+9.8% per 10 μm). Offsets in the single-digit micrometer range produced changes comparable in magnitude to meta-analytic differences reported for early diabetic microvascular impairment. Taken together, the marked device- and layer-specific offset sensitivities, often reaching effect sizes comparable to reported early diabetic microvascular differences, underscore that micrometer-scale slab definition changes can materially bias OCTA-derived endpoints. Standardized slab definitions, segmentation quality control, and transparent reporting of adjustments are therefore essential for reliable interpretation in longitudinal studies, multicenter research, and algorithm development. These quantitative sensitivity profiles may further inform practical tolerance thresholds for quality control and cross-platform harmonization in clinical studies and multicenter trials.

## Introduction

Optical coherence tomography angiography (OCTA) enables high-resolution, depth-resolved visualization of the retinal microvasculature without intravenous dye [1,2]. Since its clinical adoption, OCTA has increasingly been integrated into diagnostic workflows for retinal vascular diseases, including diabetic retinopathy, retinal vein occlusion, and age-related macular degeneration [3]. The technology’s ability to extract quantitative vascular parameters such as vessel area density (VAD), total vessel length (TVL), vessel length density (VLD), branchpoint density (BD), and foveal avascular zone (FAZ) metrics provides valuable tools for disease monitoring, prognosis, and guiding treatment [4–6]. However, the accuracy of OCTA-derived measurements heavily relies on precise layer segmentation to isolate specific vascular plexuses, primarily the superficial capillary plexus (SCP) and deep capillary plexus (DCP). Most OCTA devices produce en face images based on predefined anatomical boundaries, which, although often adjustable, remain influenced by device defaults, software updates, and user modifications. Recent research indicates that even minor deviations in segmentation can significantly impact quantitative readings, potentially confounding interpretation and obscuring actual pathological changes [7,8].

This variability becomes especially problematic in multicenter studies, long-term monitoring, and comparisons involving different OCTA devices. The issue is exacerbated by the lack of a universal agreement on the exact anatomical boundaries of the SCP and DCP, with varying manufacturers of devices employing different definitions and strategies for reducing projection artifacts [8,9]. As a result, discrepancies between devices in OCTA measurements can stem not only from hardware differences but also from inconsistent segmentation protocols. Although there is increasing research on OCTA repeatability and comparisons [10,11], a systematic evaluation of how controlled segmentation shifts affect OCTA metrics across different imaging systems has not yet been performed. Most existing studies focus on simple comparisons, such as default versus manually corrected segmentation [12,13], leaving a significant gap in understanding how minor boundary adjustments influence vascular measurements across multiple devices.

To address this methodological gap, the present study systematically examines how sensitive standard OCTA metrics are to defined segmentation boundary displacements across three commercially available devices, while ensuring consistent layer definitions and using standardized quantitative analysis. By benchmarking segmentation-induced changes against established diabetic retinopathy thresholds, we quantify the risk of misinterpreting technical artifacts as pathological changes. Our findings establish device-specific tolerance thresholds that inform quality control protocols, multicenter trial design, and algorithm development for OCTA-based diagnostics.

## Methods

### Study design and ethics

This prospective cross-sectional study received approval from the Institutional Review Board of LMU Munich (study ID: 24–0571) and was conducted in accordance with the principles of the Declaration of Helsinki. Written informed consent was obtained from all participants before any study procedures. Participants could opt out of secondary use/data sharing beyond the present study; therefore, unrestricted public release of the minimal dataset is not permitted. Data access is managed by the institutional study center under a data use agreement.

### Participants

Participants were recruited from 03-DEC-2024–02-JUN-2025 at the Department of Ophthalmology, LMU University Hospital, Munich. Inclusion criteria required two clinically healthy eyes with no history of ocular disease, best-corrected visual acuity of at least 20/25, and a spherical equivalent within ±6 diopters. Exclusion criteria included: (1) any chorioretinal pathology identified on clinical examination or optical coherence tomography; (2) systemic conditions known to affect retinal vessels, specifically arterial hypertension (blood pressure >140/90 mmHg on multiple measurements), diabetes mellitus (any type), or chronic kidney disease; (3) significant media opacities that impede high-quality imaging; (4) inability to maintain stable fixation during imaging.

### Imaging protocol

All participants underwent OCTA imaging with three devices during a single visit: Intalight DREAM Swept Source OCT VG200D (Intalight Inc., San Jose, California, USA), Zeiss Cirrus HD 5000 OCT (Carl Zeiss Meditec, Jena, Germany), and Topcon DRI-OCT Triton Swept-Source OCT (Topcon Corporation, Tokyo, Japan). The device order was randomized, with at least 5-minute intervals between scans to reduce artifacts caused by operator and participant fatigue. Pupillary dilation was not performed to preserve physiological conditions.

Each eye was imaged using the manufacturer’s native macular 3 × 3 mm OCTA scan protocol on each platform (Intalight DREAM, ZEISS Cirrus, Topcon Triton), centered on the fovea. For all devices, the scan was acquired with four repeated B-scans per location (n = 4). DREAM OCT operated at a maximum resolution of 512 × 512 pixels, while the highest resolutions available were used for Cirrus OCT (420 × 420 pixels) and Triton OCT (320 × 320 pixels).

Beyond pixel dimensions, we report each system’s manufacturer-reported optical resolution and the corresponding transverse sampling density of the 3 × 3 mm OCTA protocol, as both factors can influence OCTA image quality and quantitative metric values. DREAM OCT provides an axial resolution of 3.8 µm and a lateral resolution of 10 µm [14]; Cirrus 5 µm axial and 15 µm transverse (in tissue) [15]; and Triton 8 µm axial and 20 µm lateral [16]. For the 3 × 3 mm acquisitions used here, the sampling grids were 512 × 512 (DREAM), 420 × 420 (Cirrus), and 320 × 320 (Triton), corresponding to transverse sampling densities of 170.7, 140.0, and 106.7 samples/mm, respectively.

Only images that met predefined quality criteria were included in the analysis. Scans were considered of adequate quality only if the device-reported quality index met predefined thresholds (Intalight DREAM ≥8/10, Zeiss Cirrus ≥8/10, Topcon Triton ≥80/100) and passed standardized qualitative quality checks (no relevant motion/defocus artifacts, no major shadowing or uneven illumination, and complete visualization of the relevant retinal boundaries).

### Segmentation protocol

Standardized layer definitions were applied consistently across all three devices to ensure comparability. The SCP was defined as the region from the inner limiting membrane to the transition between the inner plexiform layer (IPL) and inner nuclear layer (INL). At the same time, the DCP extended from the IPL-INL boundary to the transition between the outer plexiform layer (OPL) and outer nuclear layer (ONL) [9]. All OCTA scans were manually reviewed on the corresponding structural OCT B-scans to confirm segmentation plausibility. Scans showing gross segmentation failure would have been excluded from analysis. The only intentional segmentation manipulation in this study was the controlled offset of the shared slab boundary used for the sensitivity experiment.

**Fig 1:**
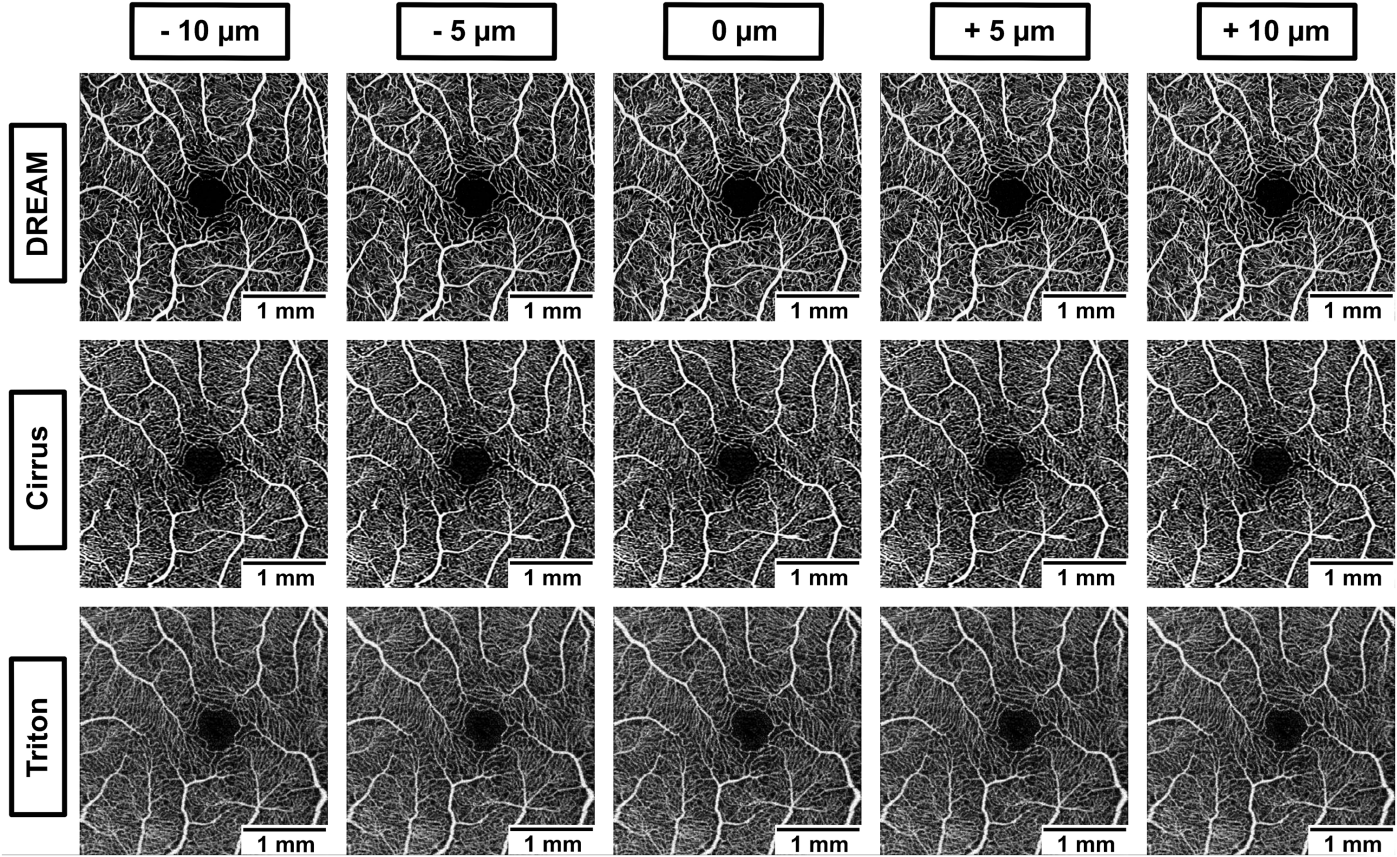
Impact of segmentation shifts on en face OCTA appearance across devices. Representative en face OCTA images of the superficial capillary plexus (SCP) from one healthy eye across three devices (DREAM, Cirrus, Triton), shown at five segmentation positions: −10 µm, −5 µm, 0 µm, + 5 µm, and +10 µm relative to a standardized anatomical baseline. Each row displays images from a different device, and each column corresponds to a specific offset.

**Fig 2:**
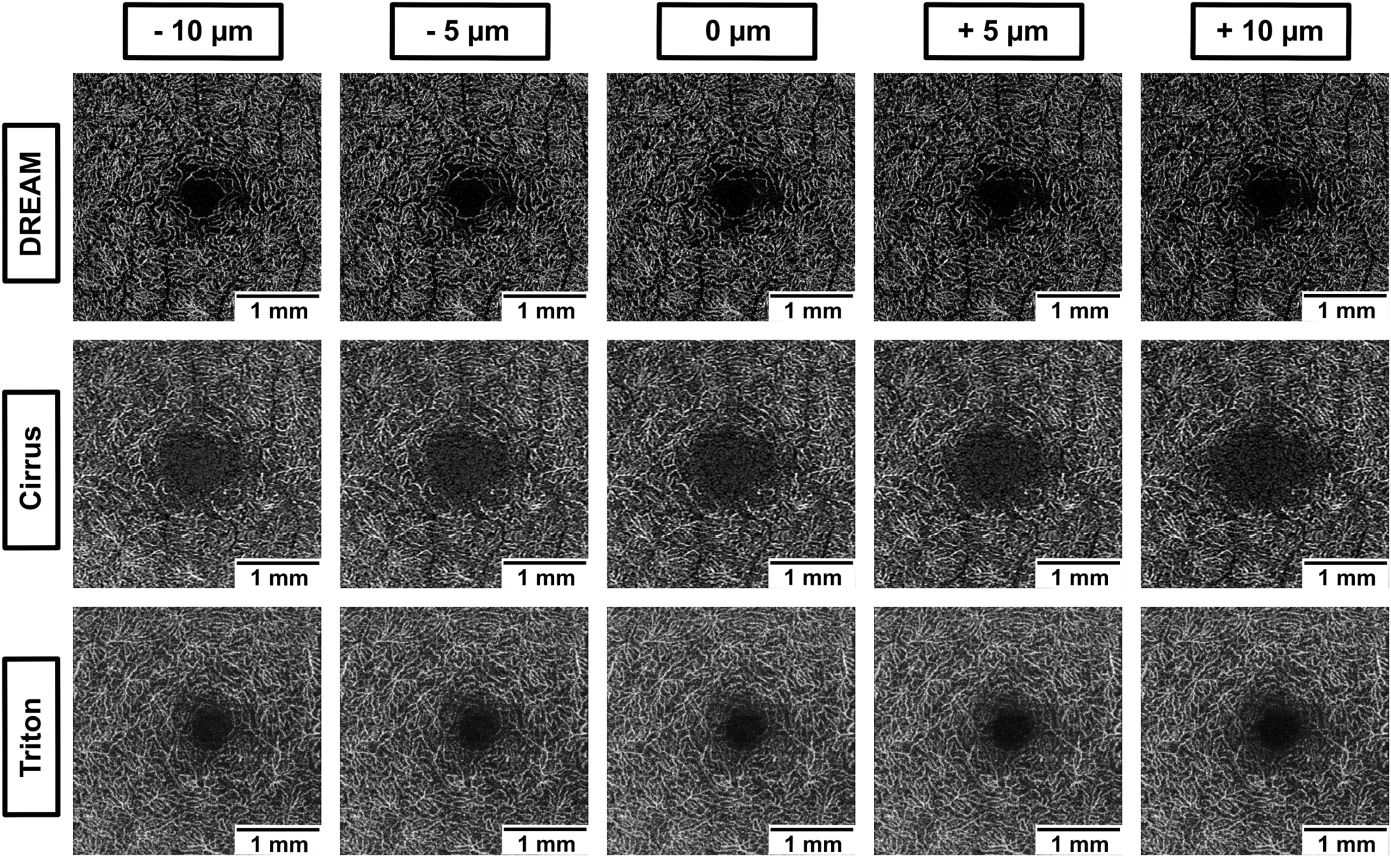
Effect of segmentation offset on DCP visualization across devices. Representative en face OCTA images of the deep capillary plexus (DCP) from one healthy eye across three devices (DREAM, Cirrus, Triton), displayed at five segmentation offsets (−10 µm to +10 µm in 5 µm steps) relative to a standardized anatomical baseline. Each row shows a different device, and the columns indicate the segmentation position.

To ensure comparability across instruments, we applied standardized anatomical slab definitions for the superficial and deep vascular layers consistently across all devices, rather than using manufacturer “default” slabs. Specifically, a superficial capillary plexus (SCP) slab and a deep capillary plexus (DCP) slab (operationally corresponding to the deep vascular complex) were defined using the same anatomical boundaries in each device’s native segmentation software. Using each device’s segmentation tools, we then generated five offset conditions by systematically shifting the shared IPL/INL boundary by −10, −5, 0, + 5, and +10 µm, while keeping the remaining boundary fixed. The 0 µm condition represents the unshifted standardized reference, and the other offsets represent controlled deviations from this reference. For each offset condition, en face OCTA maps were generated for both slabs, resulting in 10 en face maps per eye per device (5 offsets × 2 slabs). Positive values represent displacement toward the outer retinal layers. Examples of en face images for SCP are shown in Fig 1, and for DCP in Fig 2.

This figure demonstrates how systematic shifts in segmentation boundaries affect vascular visualization in the SCP. Subtle yet measurable changes in vessel density and capillary visibility are especially noticeable in the DREAM system. These device-specific variations in segmentation sensitivity highlight the risk of artifactually induced changes in quantitative OCTA metrics and emphasize the need for standardized segmentation protocols in multicenter or long-term studies.

This figure illustrates the effect of small changes to the top boundary of the DCP slab’s segmentation on the visual appearance of the capillary network. A gradual decrease in vessel density and perifoveal detail is apparent with increasing downward displacement (+µm), especially in the DREAM system. These findings align with the quantitative results and underscore the DCP’s heightened sensitivity to segmentation depth, underscoring the importance of accurate and consistent boundary placement in both clinical and research imaging processes.

### Image processing and analysis

Images were exported in a lossless format (PNG/TIFF) at the native device resolution. No additional post-hoc projection-artifact removal was applied beyond each device’s native manufacturer processing used to generate the en face slabs. Images were then preprocessed using custom Python scripts (Python 3.10.15) with the Pillow image-processing library. Prior to quantitative analysis, all images were converted to the same lossless PNG format and resampled to 512 × 512 pixels (target size corresponding to the maximum en face grid supported by DREAM) to ensure uniform pixel scaling across devices. This step was implemented to enable consistent kernel-based preprocessing (including Frangi vesselness filtering), as OCTA-derived metrics can be sensitive to filter settings and effective pixel scale [5,6].

Quantitative analysis used the open-source OCTAVA (OCT Angiography Vascular Analyzer) framework [5,6]. OCTAVA was run on MATLAB R2024b (MathWorks, Natick, USA) with standardized processing settings. A two-dimensional Frangi filter (threshold = 3) improved vessel detection by reducing background noise and intensity variations along vessels. Preprocessed images were segmented using fuzzy thresholding (adaptive threshold kernel size = 70), followed by skeletonization with the MATLAB thinning algorithm.

Network connectivity analysis involved converting the skeleton into an undirected graph, identifying branch points, and classifying vessels accordingly. Isolated elements and branches below the minimum length threshold (twig size = 2) were excluded as noise artifacts. FAZ area was obtained from automated FAZ delineation within the OCTAVA pipeline from binarized images using region-growing algorithms. To ensure comparability across devices and to mitigate known failure modes of automated FAZ segmentation, all FAZ overlays were visually reviewed by a grader for plausibility (i.e., closed contiguous contour centered at the fovea, no obvious inclusion of large vessels, and no gross boundary discontinuities). Gross segmentation failures (e.g., missing FAZ outline, clearly misplaced contour, or severe fragmentation) would have led to manual correction. In the present dataset, this quality check step ensured that FAZ estimates reflected anatomically plausible delineations prior to statistical analysis.

**Fig 3:**
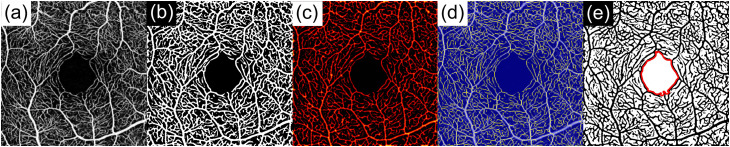
OCTAVA image-processing workflow and metric extraction for the segmentation-offset sensitivity analysis. A stepwise visualization summarizes the OCTAVA processing pipeline used for all en face OCTA slabs across devices and offset conditions.

The following microvascular metrics were extracted for both SCP and DCP: vessel area density (VAD, percentage of image area occupied by vessels), total vessel length (TVL, cumulative length of all detected vessels in mm), vessel length density (VLD, total vessel length per unit area in mm/mm^2^), number of vascular nodes (junction points), branchpoint density (BD, mm^⁻2^), and FAZ area (mm^2^). The processing and metric-extraction workflow is summarized in Fig 3.

Panel (a) shows a representative en face OCTA image (here: DREAM) after resampling to a common 512 × 512 pixel grid to ensure consistent filter scaling. Panel (b) illustrates vessel enhancement using Frangi vesselness filtering followed by binarization to obtain a vessel mask for density-based quantification. Panel (c) displays the vessel-diameter heatmap derived from a Euclidean distance transform, enabling diameter-related descriptors and supporting robust vessel characterization. Panel (d) shows the skeletonized vessel representation after thinning, which forms the basis for length- and topology-based metrics, including total vessel length (TVL), vessel length density (VLD), and network complexity descriptors such as node count and branchpoint density (BD). Panel (e) depicts automated foveal avascular zone (FAZ) delineation (red), which was used to compute FAZ area. Together, these processing steps provide a fully disclosed and reproducible workflow linking standardized preprocessing to the quantitative OCTA metrics used to evaluate sensitivity to controlled segmentation offsets (−10, −5, 0, + 5, + 10 µm).

Quantitative OCTA metrics can vary across analysis pipelines (e.g., filtering, binarization/skeletonization, and FAZ delineation), so absolute values are not necessarily interchangeable between tools [5,6]. We therefore used OCTAVA, an open-source and fully transparent workflow with explicitly reported, user-configurable preprocessing (including Frangi vesselness filtering) and metric definitions to maximize reproducibility across devices. Accordingly, our main conclusions focus on relative sensitivity patterns within a fully disclosed pipeline, rather than tool-specific absolute cutoffs.

### Statistical analysis

All analyses were performed in R (RStudio for macOS Version 2024.12.1 + 563). For each OCTA metric, separate linear mixed-effects models were fitted for each device (DREAM, Cirrus, Triton) and plexus (SCP, DCP), with segmentation offset (µm) entered as a continuous fixed-effect predictor. To account for within-participant correlation arising from inclusion of both eyes and repeated measurements across segmentation offsets, we included a participant-specific random intercept (1|Patient_ID). Model assumptions were assessed by visual inspection of residuals. Two-sided p-values <0.05 were considered statistically significant. Effect sizes are reported as the model slope (change per 1 µm), standardized slope (slope divided by the baseline standard deviation at 0 µm), and the slope-derived relative change per 10 µm expressed as a percentage of the baseline mean (0 µm). Given the exploratory aim of quantifying sensitivity patterns across devices and layers, we focused on effect sizes and consistency across metrics rather than strict family-wise error control.

An a priori power calculation was not performed because robust published estimates for micrometer-scale segmentation-offset effect sizes (slopes) across commercial OCTA platforms and metrics are currently lacking, and the primary aim of this study was exploratory (to quantify device- and layer-specific sensitivity profiles rather than to test a single prespecified hypothesis). To nevertheless provide an assessment of adequacy, we conducted a simplified, conservative sensitivity calculation based on a paired within-eye comparison between the 0 µm and +10 µm offset conditions (two-sided α = 0.05; 80% power). We used the modelled change at +10 µm (derived from the fitted slope) and the baseline SD at 0 µm as an intentionally conservative variance estimate (not accounting for the additional precision gained by the repeated-measures design with five offsets per eye). Under this approximation, the large effects observed in DREAM would require only small samples (e.g., DREAM DCP VAD: change −2.10%; SD 1.28%; n ≥ 3 eyes; DREAM SCP VAD: change +2.14%; SD 1.98%; n ≥ 7 eyes), indicating that our sample of 32 eyes is sufficient to detect the main device-specific offset effects reported for DREAM (and the larger DCP/FAZ effects). For near-zero slopes (e.g., SCP metrics in Cirrus/Triton), the same conservative calculation implies that very large samples would be required to reach statistical significance; importantly, the corresponding effect sizes were also small, supporting relative robustness to ±10 µm offsets on those platforms.

## Results

### Baseline demographics

Thirty-two eyes from 16 healthy participants (10 female, six male) with a mean age of 26.1 ± 2.9 years were successfully analyzed. All participants completed imaging with all three devices, and all acquired images met predefined quality standards, resulting in a 100% inclusion rate for analysis.

### Descriptive statistics and baseline comparisons

Descriptive statistics for all metrics across segmentation offsets, devices, and plexuses are shown in S1 Table (SCP) and S2 Table (DCP). At the default segmentation position (offset = 0 µm), notable device-dependent differences were observed in baseline vascular density and structural complexity. In the SCP, DREAM showed the highest VAD (43.69 ± 1.98%), followed by Cirrus (41.98 ± 3.27%) and Triton (33.70 ± 2.71%). Similar trends were observed for other vascular metrics, confirming known inter-device variability in absolute values [17], while the relative response patterns to segmentation shifts remained consistent.

### Superficial capillary plexus: device-specific sensitivity patterns

**Fig 4:**
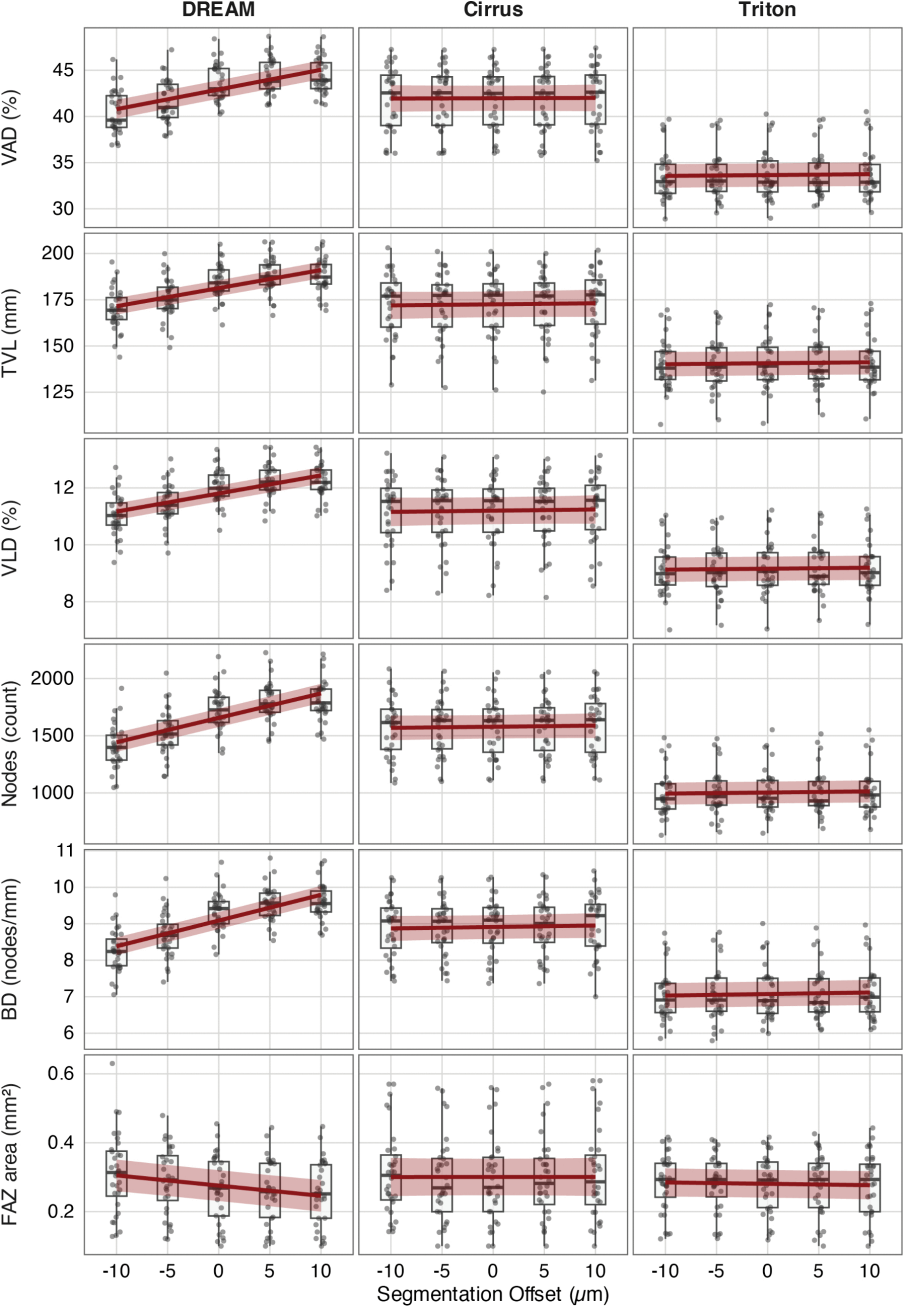
Sensitivity of superficial capillary plexus (SCP) metrics to segmentation offsets across devices. Boxplots with overlaid linear mixed-effects regression lines (red) and 95% confidence intervals (shaded area) show how segmentation boundary shifts (−10 µm to +10 µm) affect OCTA-derived SCP metrics. Metrics are arranged by row: vessel area density (VAD), total vessel length (TVL), vessel length density (VLD), node count, branchpoint density (BD), and FAZ area. Columns represent imaging devices: DREAM, Cirrus, Triton.

The DREAM system consistently showed high sensitivity to segmentation offsets across all SCP metrics. VAD increased by 0.2135%/µm (standardized slope = 0.108, p < 0.001), TVL by 0.9781 mm/µm (standardized slope = 0.106, p < 0.001), and VLD by 0.0636 (mm/mm^2^)/µm (standardized slope = 0.106, p < 0.001). Node count increased by 21.34 count/µm (standardized slope = 0.119, p < 0.001), and BD by 0.0706 nodes/mm^2^/µm (standardized slope = 0.136, p < 0.001). FAZ area significantly decreased with downward slab shifts, by −0.0030 mm^2^/µm (standardized slope = −0.030, p < 0.001), indicating progressive invasion of perifoveal capillaries. In contrast, Cirrus and Triton exhibited minimal SCP sensitivity, with no significant slope noted in any of the analyzed metrics (all p > 0.05). Data are shown in Tables 1 and 2 and visualized in Fig 4.

**Table 1:** Summary of linear mixed-effects model results. For each OCTA-derived metric and device (DREAM, Cirrus, Triton), linear mixed-effects models were fitted with segmentation offset (measured in µm) as a continuous predictor. Separate models were created for each device and vascular plexus (Superficial Capillary Plexus [SCP] and Deep Capillary Plexus [DCP]). Table entries include the model intercept, slope (i.e., effect per 1 µm offset), associated p-value, and the predicted change in the metric at +10 µm offset relative to baseline.

		device	intercept	slope (Offset)	p (slope)	∆ at +10 µm
VAD	SCP	DREAM	42.9209%	0.2135%/µm	**< 0.001**	2.135%
		Cirrus	41.9749%	0.0032%/µm	0.889	0.032%
		Triton	33.6572%	0.0095%/µm	0.311	0.095%
	DCP	DREAM	39.8467%	−0.2099%/µm	**< 0.001**	−2.099%
		Cirrus	37.9238%	−0.0776%/µm	**0.001**	−0.776%
		Triton	38.4605%	−0.0052%/µm	0.639	−0.052%
TVL	SCP	DREAM	181.3046 mm	0.9781 mm/µm	**< 0.001**	9.781 mm
		Cirrus	172.5357 mm	0.0578 mm/µm	0.634	0.578 mm
		Triton	140.6853 mm	0.0523 mm/µm	0.326	0.523 mm
	DCP	DREAM	177.5333 mm	−0.9054 mm/µm	**< 0.001**	−9.054 mm
		Cirrus	160.5626 mm	−0.3257 mm/µm	**< 0.001**	−3.257 mm
		Triton	164.9385 mm	−0.0444 mm/µm	0.478	−0.444 mm
VLD	SCP	DREAM	11.8039 mm/mm²	0.0636 (mm/mm²)/µm	**< 0.001**	0.636 mm/mm²
		Cirrus	11.1991 mm/mm²	0.0042 (mm/mm²)/µm	0.617	0.042 mm/mm²
		Triton	9.1593 mm/mm²	0.0034 (mm/mm²)/µm	0.329	0.034 mm/mm²
	DCP	DREAM	11.5583 mm/mm²	−0.0589 (mm/mm²)/µm	**< 0.001**	−0.589 mm/mm²
		Cirrus	10.4533 mm/mm²	−0.0212 (mm/mm²)/µm	**< 0.001**	−0.212 mm/mm²
		Triton	10.7383 mm/mm²	−0.0029 (mm/mm²)/µm	0.477	−0.029 mm/mm²
Nodes	SCP	DREAM	1656.1375	21.3406 count/µm	**< 0.001**	213.406
		Cirrus	1578.3000	0.9756 count/µm	0.562	9.756
		Triton	1003.5688	0.9663 count/µm	0.173	9.663
	DCP	DREAM	1695.4938	−18.3669 count/µm	**< 0.001**	−183.669
		Cirrus	1469.3875	−2.5863 count/µm	0.051	−25.863
		Triton	1511.0563	−0.6950 count/µm	0.399	−6.950
BD	SCP	DREAM	9.0871 mm^-2^	0.0706 mm^-2^/µm	**< 0.001**	0.706 mm^-2^
		Cirrus	8.9103 mm^-2^	0.0040 mm^-2^/µm	0.452	0.040 mm^-2^
		Triton	7.0730 mm^-2^	0.0042 mm^-2^/µm	0.128	0.042 mm^-2^
	DCP	DREAM	9.5236 mm^-2^	−0.0560 mm^-2^/µm	**< 0.001**	−0.560 mm^-2^
		Cirrus	9.0964 mm^-2^	−0.0026 mm^-2^/µm	0.498	−0.026 mm^-2^
		Triton	9.1541 mm^-2^	−0.0016 mm^-2^/µm	0.503	−0.016 mm^-2^
FAZ	SCP	DREAM	0.2756 mm²	−0.0030 mm²/µm	**< 0.001**	−0.030 mm²
		Cirrus	0.3006 mm²	0.0000 mm²/µm	0.989	0.000 mm²
		Triton	0.2808 mm²	−0.0004 mm²/µm	0.322	−0.004 mm²
	DCP	DREAM	0.3393 mm²	0.0051 mm²/µm	**< 0.001**	0.051 mm²
		Cirrus	0.7367 mm²	0.0116 mm²/µm	**< 0.001**	0.116 mm²
		Triton	0.5230 mm²	0.0050 mm²/µm	**< 0.001**	0.050 mm²

Abbreviations: VAD = vessel area density, TVL = total vessel length, VLD = vessel length density, BD = branchpoint density, FAZ = foveal avascular zone.

**Table 2:** Standardized sensitivity of OCTA-derived metrics to segmentation offset across devices and plexuses. This table summarizes the linear slope, standardized slope, and slope-derived relative change per 10 µm of six OCTA-derived microvascular parameters: vessel area density (VAD), total vessel length (TVL), vessel length density (VLD), number of vascular nodes, branchpoint density (BD), and foveal avascular zone (FAZ) area. These are analyzed across three commercial OCTA systems (DREAM, Cirrus, Triton) and two vascular layers (superficial capillary plexus [SCP] and deep capillary plexus [DCP]). The slope indicates the modeled change in the respective metric for each 1 µm segmentation offset, as estimated by linear mixed-effects modeling. The standardized slope is obtained by dividing the absolute slope by the standard deviation (SD) of the corresponding metric at a 0 µm offset, enabling comparison of sensitivity across different metrics and units. The slope-derived relative change per 10 µm (%) measures the slope as a percentage of the average value of that metric, indicating the percentage change in the parameter for each 10 µm displacement. This offers an intuitive understanding of how sensitive each metric is to segmentation shifts relative to its typical size. Higher values suggest greater vulnerability to segmentation-related variability, which could affect the robustness of metrics in longitudinal or multi-device studies.

		device	slope	mean	SD	standardized slope	Slope-derived relative change per 10 µm (%)
VAD	SCP	DREAM	0.2135%/µm	43.69%	1.98%	0.108	4.89
		Cirrus	0.0032%/µm	41.98%	3.27%	0.001	0.08
		Triton	0.0095%/µm	33.70%	2.71%	0.004	0.28
	DCP	DREAM	−0.2099%/µm	40.52%	1.28%	−0.164	−5.18
		Cirrus	−0.0776%/µm	38.03%	3.73%	−0.021	−2.04
		Triton	−0.0052%/µm	38.49%	1.41%	−0.004	−0.14
TVL	SCP	DREAM	0.9781 mm/µm	184.81 mm	9.24 mm	0.106	5.29
		Cirrus	0.0578 mm/µm	172.35 mm	17.26 mm	0.003	0.34
		Triton	0.0523 mm/µm	141.02 mm	13.93 mm	0.004	0.37
	DCP	DREAM	−0.9054 mm/µm	180.55 mm	7.24 mm	−0.125	−5.01
		Cirrus	−0.3257 mm/µm	161.11 mm	14.66 mm	−0.022	−2.02
		Triton	−0.0444 mm/µm	165.00 mm	9.63 mm	−0.005	−0.27
VLD	SCP	DREAM	0.0636 (mm/mm²)/µm	12.03 (mm/mm²)	0.60 (mm/mm²)	0.106	5.29
		Cirrus	0.0042 (mm/mm²)/µm	11.17 (mm/mm²)	1.20 (mm/mm²)	0.004	0.38
		Triton	0.0034 (mm/mm²)/µm	9.18 (mm/mm²)	0.91 (mm/mm²)	0.004	0.37
	DCP	DREAM	−0.0589 (mm/mm²)/µm	11.76 (mm/mm²)	0.47 (mm/mm²)	−0.125	−5.01
		Cirrus	−0.0212 (mm/mm²)/µm	10.49 (mm/mm²)	0.95 (mm/mm²)	−0.022	−2.02
		Triton	−0.0029 (mm/mm²)/µm	10.74 (mm/mm²)	0.63 (mm/mm²)	−0.005	−0.27
Nodes	SCP	DREAM	21.3406 count/µm	1728.25	179.93	0.119	12.35
		Cirrus	0.9756 count/µm	1571.94	247.56	0.004	0.62
		Triton	0.9663 count/µm	1010.06	203.93	0.005	0.96
	DCP	DREAM	−18.3669 count/µm	1753.78	122.18	−0.150	−10.47
		Cirrus	−2.5863 count/µm	1476.13	243.49	−0.011	−1.75
		Triton	−0.6950 count/µm	1503.38	113.94	−0.006	−0.46
BD	SCP	DREAM	0.0706 mm^-2^/µm	9.33 mm^-2^	0.52 mm^-2^	0.136	7.57
		Cirrus	0.0040 mm^-2^/µm	8.90 mm^-2^	0.77 mm^-2^	0.005	0.45
		Triton	0.0042 mm^-2^/µm	7.10 mm^-2^	0.74 mm^-2^	0.006	0.59
	DCP	DREAM	−0.0560 mm^-2^/µm	9.70 mm^-2^	0.35 mm^-2^	−0.160	−5.77
		Cirrus	−0.0026 mm^-2^/µm	9.10 mm^-2^	0.71 mm^-2^	−0.004	−0.29
		Triton	−0.0016 mm^-2^/µm	9.10 mm^-2^	0.26 mm^-2^	−0.006	−0.18
FAZ	SCP	DREAM	−0.0030 mm²/µm	0.26 mm²	0.10 mm²	−0.030	−11.54
		Cirrus	0.0000 mm²/µm	0.29 mm²	0.13 mm²	0.000	0.00
		Triton	−0.0004 mm²/µm	0.29 mm²	0.10 mm²	−0.004	−1.38
	DCP	DREAM	0.0051 mm²/µm	0.33 mm²	0.12 mm²	0.042	15.45
		Cirrus	0.0116 mm²/µm	0.72 mm²	0.26 mm²	0.045	16.11
		Triton	0.0050 mm²/µm	0.51 mm²	0.17 mm²	0.029	9.80

This figure illustrates that the DREAM system exhibits strong positive correlations between segmentation offset and all SCP metrics, characterized by steeper regression slopes and clear upward trends. In contrast, Cirrus and Triton show little to no offset-related change across all parameters. The FAZ area has a slight negative offset sensitivity in DREAM but remains stable in Cirrus and Triton. These results support the conclusion that SCP-derived vascular metrics, especially from DREAM, are affected by even minor segmentation deviations, which could weaken metric stability in clinical and research settings without standardized slab definitions.

### Deep capillary plexus: amplified sensitivity across devices

In the DREAM system, all DCP metrics showed strong correlations with segmentation depth. VAD decreased by −0.2099%/µm (standardized slope = −0.164, p < 0.001), TVL by −0.9054 mm/µm (standardized slope = −0.125, p < 0.001), and VLD by −0.0589 (mm/mm^2^)/µm (standardized slope = −0.125, p < 0.001). Node count dropped by −18.37 count/µm (standardized slope = −0.150, p < 0.001), and BD by −0.0560 mm^-2^/µm (standardized slope = −0.160, p < 0.001). FAZ area increased significantly, by +0.0051 mm^2^/µm (standardized slope = 0.042, p < 0.001).

Cirrus showed moderate DCP sensitivity. VAD decreased by −0.0776%/µm (standardized slope = −0.021, p = 0.001), TVL decreased by −0.3257 mm/µm (standardized slope = −0.022, p < 0.001), and VLD dropped by −0.0212 (mm/mm^2^)/µm (standardized slope = −0.022, p < 0.001). Node count showed a borderline significant trend (−2.59/µm, p = 0.051), while BD was unaffected (−0.0026 mm ⁻ ^2^/µm, p = 0.498). FAZ area increased sharply by +0.0116 mm^2^/µm (standardized slope = 0.045, p < 0.001), indicating the highest relative FAZ sensitivity among all devices.

**Fig 5:**
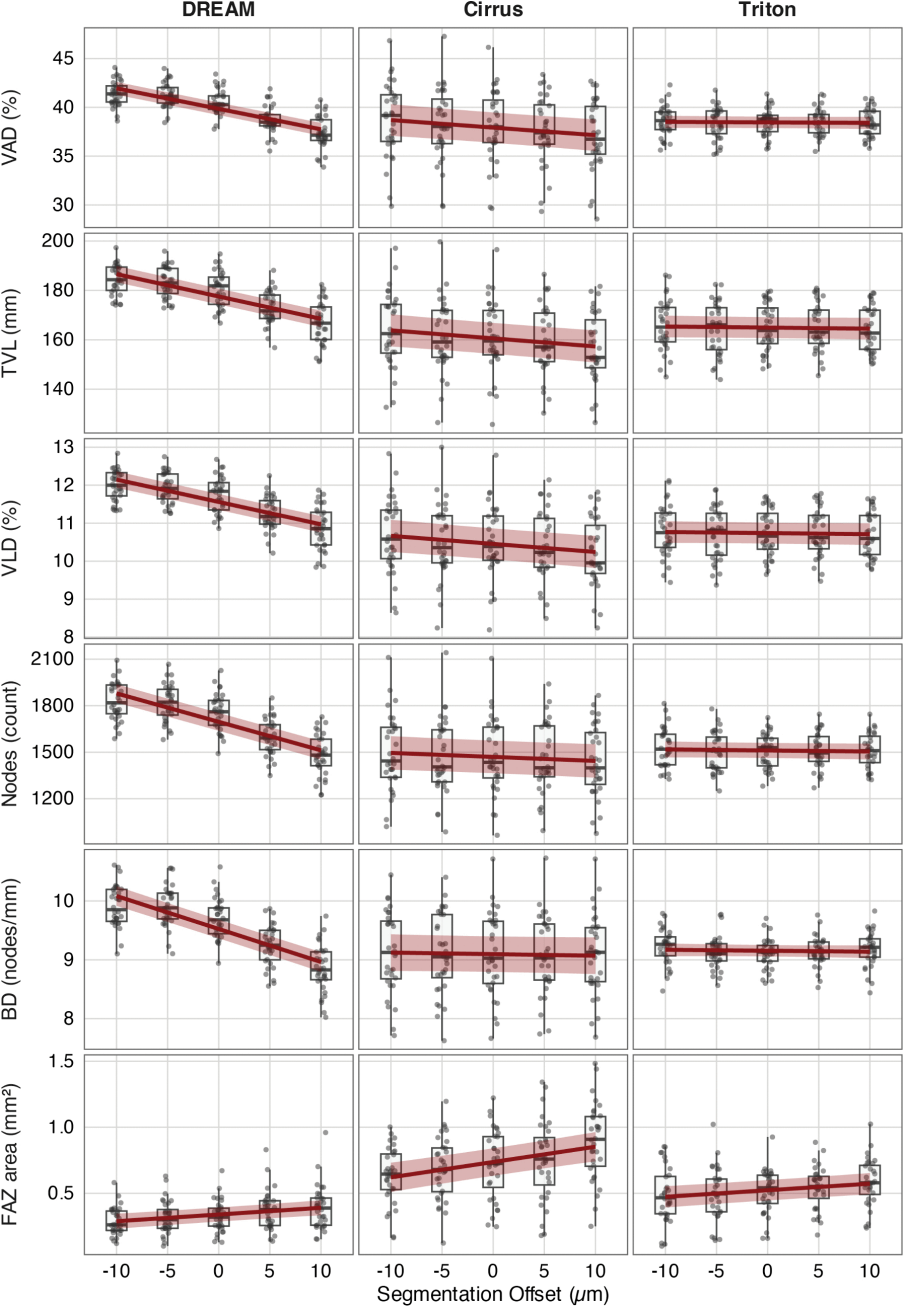
Sensitivity of deep capillary plexus (DCP) metrics to segmentation offsets across devices. Boxplots with overlaid linear mixed-effects regression lines (red) and 95% confidence intervals (shaded area) for DCP metrics at segmentation offsets ranging from −10 µm to +10 µm. Metrics, from top to bottom, include vessel area density (VAD), total vessel length (TVL), vessel length density (VLD), node count, branchpoint density (BD), and FAZ area. Columns represent imaging systems: DREAM, Cirrus, and Triton.

Triton demonstrated high DCP stability across vascular metrics. VAD declined only slightly (−0.0052%/µm; standardized slope = −0.004, p = 0.639), TVL by −0.0444 mm/µm (standardized slope = −0.005, p = 0.478), and VLD by −0.0029 (mm/mm^2^)/µm (standardized slope = −0.005, p = 0.477). Node count and BD showed minimal changes (both p > 0.3). FAZ area, however, remained sensitive, increasing by +0.0050 mm^2^/µm (standardized slope = 0.029, p < 0.001), confirming selective metric vulnerability. Data are presented in Tables 1 and 2 and visualized in Fig 5.

This figure illustrates an apparent decline in vascular density and structural complexity within the DREAM system as the DCP segmentation slab is moved downward, corresponding to the exclusion of intermediate plexus capillaries. Cirrus demonstrates moderate sensitivity to offset, especially in the VAD and FAZ regions, while Triton metrics stay mostly unchanged. Notably, the FAZ area increases with offset in both DREAM and Cirrus, reaching values similar to those of early diabetic retinopathy, with a displacement of just ±10 µm. These results emphasize the sensitivity of DCP metrics, especially the FAZ area, to small segmentation shifts and highlight the importance of standardized, reproducible segmentation protocols across devices.

### Benchmarking segmentation-induced changes against published effect sizes

To evaluate the clinical significance of segmentation-induced metric changes, we compared the observed slopes to established diabetic retinopathy thresholds from meta-analytic data. In the SCP, DREAM reached the VAD threshold associated with early diabetic changes (absolute −1.99% [18]) at an offset of −9.3 µm (upward). For VLD, the diabetic reference threshold (−0.75 mm/mm^2^ [18]) was reached at an offset of −11.8 µm. In contrast, Cirrus and Triton required segmentation shifts exceeding −180 µm (extrapolated beyond the tested range) to replicate these changes. The FAZ area in the SCP showed relevant sensitivity only in DREAM, where an offset of −10.0 µm (upward) resulted in an enlargement of +0.03 mm^2^, which aligns with the diabetic threshold reported by [18]. Triton needed −75.0 µm (upward) to reach this value, while Cirrus did not exceed the threshold even at maximum displacement.

In the DCP, all three devices showed increased sensitivity. The offset required to match the magnitude of the reported mean change (absolute −1.42% [18]) was at 6.8 µm (downward) for DREAM, while Cirrus required 18.3 µm (downward), and Triton needed 273.1 µm (downward). The FAZ area in the DCP exceeded the diabetic enlargement threshold (+0.07 mm^2^ [18]) in all systems: DREAM at 13.7 µm (downward), Cirrus at 6.0 µm (downward), and Triton at 14.0 µm (downward). The results of this analysis are shown in Table 3.

**Table 3:** Segmentation-induced changes benchmarked against published OCTA effect sizes reported for early diabetic retinopathy. For each OCTA metric, the table displays the typical pathological changes observed in early diabetic retinopathy (DR) based on meta-analytic evidence [18], along with the segmentation offset (in µm) required to reproduce an equivalent change in each device (DREAM, Cirrus, Triton). This table directly illustrates the clinical importance of segmentation variability by showing how small shifts in slab boundaries can imitate disease-related changes in key OCTA parameters. These results highlight that minor segmentation errors can lead to false-positive or false-negative findings in both research and clinical OCTA evaluations. Linear slopes were calculated within ±10 µm, where a first-order approximation is appropriate; larger offsets may deviate from the actual nonlinear relationship but still help estimate clinical significance. Devices like DREAM, which are more affected by boundary shifts, require rigorous segmentation control to ensure accurate measurements. Asterisked (*) values generally fall outside the ± 10 µm range, are extrapolated estimates, and are unrealistically high for clinical use, indicating low sensitivity to offsets in the respective system.

Metric	Typical Pathological Change in DR (values from [18])	Offset DREAM	Offset Cirrus	Offset Triton
VAD (SCP)	−1.99%; [−2.76, −1.22] %	−9.3 µm	−621.9 µm *	−209.5 µm *
VAD (DCP)	−1.42%; [−2.32, −0.52] %	6.8 µm	18.3 µm *	273.1 µm *
VLD (SCP)	−0.75 mm/mm²; [−1.88, 0.38] mm/mm²	−11.8 µm	−178.6 µm *	−220.6 µm *
FAZ Area (SCP)	0.03 mm^2^; [0.01, 0.05] mm^2^	−10.0 µm	- ∞ µm *	−75.0 µm *
FAZ Area (DCP)	0.07 mm^2^; [0.03, 0.12] mm^2^	13.7 µm	6.0 µm	14.0 µm

### Cross-device sensitivity comparison

**Fig 6:**
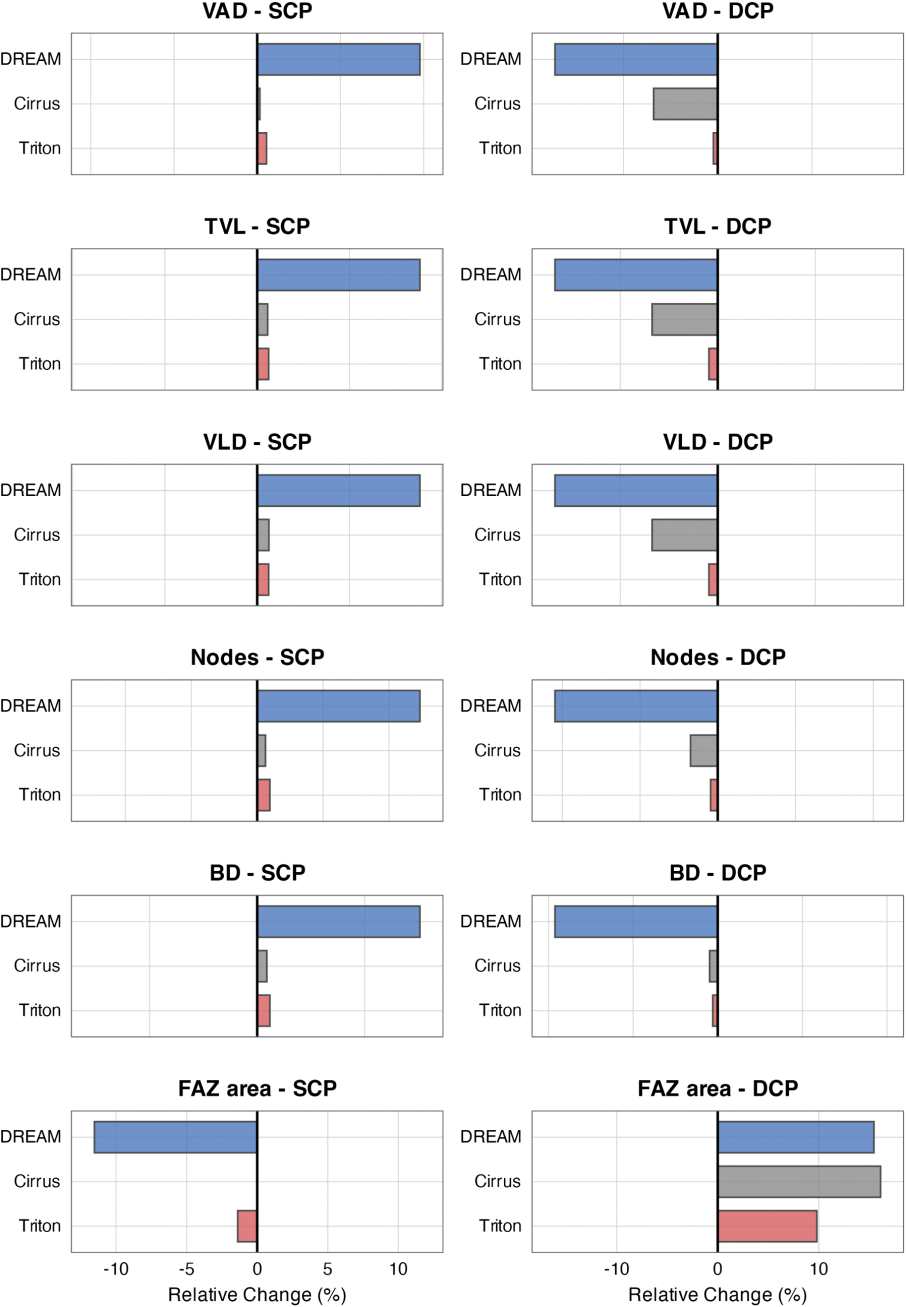
Device-specific sensitivity of OCTA metrics to segmentation shifts, expressed as slope-derived relative change per 10 µm (%). Bar plots display the slope-derived relative change per 10 µm segmentation offset for each metric and device in the superficial (SCP, left column) and deep capillary plexus (DCP, right column). Compared to Cirrus and Triton, the DREAM system consistently showed higher sensitivity across most metrics in both plexuses. Colors represent devices: DREAM (blue), Cirrus (grey), and Triton (red). Abbreviations: VAD = vessel area density, TVL = total vessel length, VLD = vessel length density, BD = branchpoint density, FAZ = foveal avascular zone.

A direct comparison of device-specific sensitivity profiles showed a clear hierarchy across all metrics. DREAM consistently exhibited the highest susceptibility to segmentation shifts. In the SCP, slope-derived relative change per 10 µm reached +4.9% for VAD, + 5.3% for TVL and VLD, + 12.4% for node count, and +7.6% for BD. In the DCP, VAD decreased by 5.2%, and the FAZ area increased by 15.5%. Cirrus demonstrated moderate sensitivity, mainly in the DCP. Here, VAD, VLD, and TVL each decreased by approximately 2.0%, and the FAZ area increased by 16.1%. In the SCP, Cirrus metrics remained stable, with all slope-derived relative changes per 10 µm being less than 0.6%. Triton showed the most consistent behavior across both layers. Slope-derived relative change per 10 µm in vascular density and branching metrics stayed below 1.0%. The only exception was the FAZ area in the DCP, which increased by +9.8% per 10 µm shift. Results are shown in Table 2 and visually summarized in Fig. 6.

## Discussion

This study provides a quantitative cross-device estimate of how small segmentation boundary offsets propagate into commonly reported OCTA metrics. By applying a harmonized slab definition and controlling ±10 μm displacement across three commercial systems, we provide strong evidence of significant device-dependent variability, mainly affecting deep plexus and FAZ metrics. These findings have direct implications for clinical interpretation, long-term follow-up, the design of multicenter studies, and algorithmic OCTA analysis.

### Device-dependent sensitivity mechanisms

The notable differences in metric sensitivity observed between systems likely originate from a combination of hardware specifications, segmentation algorithms, and image processing methods. The DREAM system, which features high axial resolution and swept-source technology, showed consistently high sensitivity in both plexuses. Even small offset changes (e.g., ± 5 μm) significantly affected SCP and DCP vascular metrics, indicating that DREAM’s high vessel detection sensitivity may reduce robustness to segmentation variability.

Cirrus, based on spectral-domain OCT, exhibited intermediate sensitivity, particularly in DCP and FAZ measurements. This may indicate limited axial resolution and layer definition accuracy in the deeper retinal layers. Triton, although also a swept-source system, showed generally low offset sensitivity, suggesting effective interpolation and boundary smoothing. Nonetheless, the FAZ area remained vulnerable across all systems, highlighting this metric’s overall susceptibility to segmentation artifacts, possibly due to its proximity to inter-plexus transitional capillaries.

Although DREAM provides high nominal image quality, it also exhibited the steepest segmentation-offset sensitivity. A plausible explanation is that, in our 3 × 3 mm protocol, DREAM combines high optical resolution (manufacturer-reported 10 µm lateral and 3.8 µm axial) with the densest transverse sampling (512 × 512; 170.7 samples/mm; 5.86 µm/sample). This configuration enhances the detectability of fine perifoveal capillary structure, including capillary segments located immediately adjacent to the shared IPL/INL interface used to define the superficial versus deep slab. Consequently, small systematic boundary shifts (±5–10 µm) can include or exclude a comparatively large amount of vascular signal in this transition zone, yielding larger changes in density- and length-based metrics and FAZ readouts than in more coarsely sampled acquisitions. In addition, device-specific processing characteristics (e.g., projection-artifact handling and en face rendering) may modulate the amount of mixed-plexus signal near the slab interface and thereby influence the observed segmentation sensitivity profile. Importantly, we interpret DREAM’s larger slopes not as inferior segmentation performance per se, but as an expected consequence of a system that resolves and samples microvascular structure more finely, making quantitative outputs intrinsically more responsive to micrometer-scale slab-definition changes.

### Clinical relevance and risk of diagnostic misinterpretation

The magnitude of segmentation-induced metric changes observed in our study has direct clinical relevance. For instance, a downward shift of only 6.8 μm in the DCP slab on the DREAM system replicated the mean VAD reduction reported for early diabetic retinopathy (−1.42%) [18]. Similarly, FAZ area changes exceeded the diabetic threshold (+0.07 mm^2^) on all devices with offsets between 6–14 μm.

These findings highlight how minor, unnoticed segmentation differences, caused by software updates, operator adjustments, or physiological variability, can produce changes of a magnitude comparable to those reported for early microvascular alterations. This is especially important in clinical follow-up and treatment monitoring, where longitudinal OCTA metrics help guide therapy choices. Without consistent slab definitions, even automated systems may show misleading trends that are due to technical artifacts rather than fundamental biological changes.

Junction points (nodes) and branchpoint density are best interpreted as network-complexity descriptors derived from skeleton/graph analyses, which may capture aspects of capillary remodeling (e.g., altered connectivity) and complement density-based OCTA endpoints in research settings. Because clinical interpretability and disease-specific thresholds for branching metrics are currently less standardized than for vessel density or FAZ measures, we treat nodes/BD primarily as supportive readouts for sensitivity analyses and processing/quality-control robustness [5,19].

### Benchmarking approach and clinical context

Our comparison with diabetic retinopathy thresholds requires careful interpretation. While our healthy cohort precludes direct clinical validation, this benchmarking serves two critical purposes: First, it provides an intuitive clinical reference scale for the magnitude of segmentation-induced changes, making the technical variability comprehensible to clinicians who regularly interpret OCTA findings. Second, and more importantly, it demonstrates that artifactual changes can equal or exceed well-established pathological signals, directly illustrating the risk of misinterpretation when segmentation quality is not rigorously controlled. The fact that offsets in the single-digit micrometer range can replicate disease-associated changes underscores the urgency of implementing standardized protocols, particularly in settings where OCTA metrics inform clinical decisions or serve as trial endpoints.

### Implications for multicenter research and AI development

The quantified device-specific sensitivity profiles presented here reveal a significant challenge for multicenter trials and the pooling of retrospective data. Even when using standardized image analysis pipelines like OCTAVA [5,6], segmentation-induced variability may create systematic bias that exceeds the magnitude of the biological signal, especially in early-stage disease. This is particularly problematic for vascular metrics in the DCP and FAZ, which are often used as imaging endpoints in clinical studies on diabetic retinopathy and macular ischemia.

Artificial intelligence (AI) algorithms trained on diverse datasets may unintentionally learn device- or segmentation-specific noise patterns if slab variability is not adequately controlled or modeled. Including segmentation uncertainty in model development—such as through data augmentation, uncertainty-aware loss functions, or multi-slab ensemble strategies—plays a crucial role in enhancing model generalizability and interpretability.

### Comparison with previous literature

Our findings expand upon and quantify previous observations regarding the impact of segmentation on OCTA metrics. Rommel et al. reported that the FAZ area is affected by segmentation corrections in healthy adults, consistent with our overall finding of FAZ vulnerability [20]. Ghasemi Falavarjani et al. showed that metric changes occur after segmentation error correction in both healthy and diabetic eyes, supporting our concerns about the reliability of clinical interpretations [13].

However, our study goes beyond previous binary comparison methods by offering continuous sensitivity measurements across multiple devices and directly relating them to clinical thresholds. The systematic offset approach enables the accurate estimation of segmentation tolerance limits and unique device vulnerability profiles that were previously unavailable.

Sampson et al. highlighted the effects of software updates on OCTA metrics, supporting our findings regarding systematic boundary definition changes [7]. Our results provide a quantitative framework for evaluating variations in software-related metrics and establishing effective control procedures.

### Limitations and future directions

Our controlled design in healthy eyes enabled precise quantification of technical variability without confounding by pathological features that complicate segmentation. This approach establishes baseline sensitivity profiles that define the measurement uncertainty inherent to each device, regardless of disease state. The device-specific tolerance thresholds we derived are directly applicable to quality control systems in both research and clinical settings.

Several aspects warrant further investigation. First, our linear sensitivity models are valid within the ± 10 µm range we systematically tested; larger offsets may exhibit nonlinear behavior, though such extreme misalignments would typically be apparent on visual inspection. Second, while we did not assess inter-operator variability in manual corrections, our findings quantify the magnitude of error that such variability can introduce, emphasizing the need for automated verification tools. Third, extending this analysis to eyes with pathology, where segmentation is more challenging, would provide valuable insights into disease-specific vulnerability patterns and help refine quality control thresholds for specific clinical applications. Moreover, intra-device repeatability was not assessed.

When comparing absolute OCTA-derived metrics across devices, ocular magnification should be considered: although commercial instruments report a nominal 3 × 3 mm scan, the true retinal area imaged varies with individual axial length, and most commercial OCTA systems do not automatically correct area- or density-based outputs for this effect [21]. Uncorrected axial-length variation can therefore bias FAZ area and vessel density/length measures and contribute to apparent inter-device differences in absolute values [22]. Importantly, this limitation is unlikely to affect our primary inference, because our main endpoint was the within-device change across controlled segmentation offsets applied to the same acquisition (offset-response slopes), which should be comparatively less sensitive to magnification than cross-device comparisons of single-slab absolute metrics.

Future work should investigate how projection artifact removal algorithms interact with segmentation variability, add dedicated within-device repeatability experiments to disentangle acquisition variability from processing sensitivity, evaluate the effectiveness of automated quality control systems based on our sensitivity thresholds, and assess whether device-specific correction factors can harmonize metrics across platforms in multicenter studies.

### Evidence-based recommendations for clinical practice and research

Our quantified sensitivity profiles enable specific, actionable recommendations:

**For clinical practice:** (1) Implement automated segmentation verification with device-specific tolerance thresholds (e.g., < 7 µm for DREAM DCP metrics, < 20 µm for Cirrus DCP, < 15 µm for FAZ measurements on all devices). (2) Prioritize visual inspection and manual correction for DCP and FAZ measurements, which showed universal sensitivity across devices. (3) When monitoring treatment response or disease progression, maintain consistent segmentation protocols across visits and flag any boundary adjustments that exceed device-specific tolerance thresholds.

**For research applications:** (1) Report complete segmentation parameters in all studies using quantitative OCTA metrics, including slab definitions, software versions, and any manual adjustments performed. (2) In multicenter trials, implement centralized segmentation quality control with predetermined tolerance limits based on device-specific sensitivity profiles. (3) Incorporate segmentation uncertainty into statistical power calculations, recognizing that technical variability may equal or exceed biological signals in early disease. (4) Consider preferentially using metrics with demonstrated low offset sensitivity (e.g., Triton SCP metrics) when segmentation quality cannot be rigorously controlled.

**For algorithm development:** AI models trained on OCTA data should incorporate segmentation variability through data augmentation or uncertainty-aware training to avoid learning device- or segmentation-specific artifacts. Our device-specific sensitivity profiles can guide the design of synthetic offset perturbations that improve model robustness and generalizability across imaging platforms.

## Conclusions

This systematic cross-device analysis demonstrates that segmentation offsets within ±10 µm can produce metric changes comparable in magnitude to early diabetic microvascular impairment, with pronounced device- and layer-specific vulnerability patterns. The DREAM system showed the highest sensitivity across both plexuses, while FAZ measurements were universally sensitive across all devices tested. These quantified sensitivity profiles establish evidence-based tolerance thresholds for quality control implementation in clinical practice and provide a framework for standardizing OCTA metrics in multicenter research. As OCTA increasingly informs treatment decisions and serves as a trial endpoint, rigorous segmentation verification, guided by device-specific sensitivity profiles, is essential for ensuring that measured changes reflect true biological signals rather than technical artifacts. Our findings provide the quantitative foundation for developing automated quality control systems and harmonization strategies that will support the continued integration of OCTA into evidence-based ophthalmology.

## Supporting information

S1 Table Descriptive statistics of OCTA metrics across segmentation offsets and devices in superficial capillary plexus. For each combination of device (DREAM, Cirrus, Triton) and segmentation offset (−10 µm to +10 µm), descriptive statistics are provided for six OCTA-derived metrics: vessel area density (VAD), total vessel length (TVL), vessel length density (VLD), number of vascular nodes, branchpoint density (BD), and foveal avascular zone (FAZ) area. Metrics are summarized by median, first quartile (Q25), third quartile (Q75), interquartile range (IQR = Q75 − Q25), mean, and standard deviation (SD).
(DOCX)

S2 Table Descriptive statistics of OCTA metrics across segmentation offsets and devices in deep capillary plexus. For each combination of device (DREAM, Cirrus, Triton) and segmentation offset (−10 µm to +10 µm), descriptive statistics are provided for six OCTA-derived metrics: vessel area density (VAD), total vessel length (TVL), vessel length density (VLD), number of vascular nodes, branchpoint density (BD), and foveal avascular zone (FAZ) area. Metrics are summarized by median, first quartile (Q25), third quartile (Q75), interquartile range (IQR = Q75 − Q25), mean, and standard deviation (SD).
(DOCX)
