# Supplementary material for: Quantifying segmentation sensitivity in OCTA: Device-specific profiles across three commercial platforms
Source: PLoS One. 2026 Feb 27;21(2):e0343605. doi: 10.1371/journal.pone.0343605 (PMC12948080; doi:10.1371/journal.pone.0343605)
Supplement: S2 Table — For each combination of device (DREAM, Cirrus, Triton) and segmentation offset (−10 µm to +10 µm), descriptive statistics are provided for six OCTA-derived metrics: vessel area density (VAD), total vessel length (TVL), vessel length density (VLD), number of vascular nodes, branchpoint density (BD), and foveal avascular zone (FAZ) area. Metrics are summarized by median, first quartile (Q25), third quartile (Q75), interquartile range (IQR = Q75 − Q25), mean, and standard deviation (SD). (DOCX) [file pone.0343605.s002.docx]

**S2 Table. Descriptive statistics of OCTA metrics across segmentation offsets and devices in deep capillary plexus**

*For each combination of device (DREAM, Cirrus, Triton) and segmentation offset (−10 µm to +10 µm), descriptive statistics are provided for six OCTA-derived metrics: vessel area density (VAD), total vessel length (TVL), vessel length density (VLD), number of vascular nodes, branchpoint density (BD), and foveal avascular zone (FAZ) area. Metrics are summarized by median, first quartile (Q25), third quartile (Q75), interquartile range (IQR = Q75 − Q25), mean, and standard deviation (SD).*

|  |  |  | **variable** | **median** | **Q25** | **Q75** | **IQR** | **mean** | **SD** |
| --- | --- | --- | --- | --- | --- | --- | --- | --- | --- |
| DCP | DREAM | -10 | VAD (%) | 41.37 | 40.54 | 42.20 | 1.65 | 41.37 | 1.27 |
|  |  |  | TVL (mm) | 184.34 | 179.99 | 189.44 | 9.45 | 184.30 | 6.14 |
|  |  |  | VLD (mm/mm²) | 12.00 | 11.72 | 12.33 | 0.61 | 12.00 | 0.40 |
|  |  |  | Nodes (count) | 1818.50 | 1748.00 | 1933.25 | 185.25 | 1828.59 | 122.73 |
|  |  |  | BD (mm^-2^) | 9.85 | 9.65 | 10.20 | 0.54 | 9.91 | 0.36 |
|  |  |  | FAZ area (mm²) | 0.26 | 0.22 | 0.37 | 0.15 | 0.29 | 0.10 |
|  |  | -5 | VAD (%) | 41.11 | 40.44 | 42.04 | 1.60 | 41.25 | 1.24 |
|  |  |  | TVL (mm) | 182.98 | 178.76 | 188.88 | 10.13 | 183.17 | 6.21 |
|  |  |  | VLD (mm/mm²) | 11.92 | 11.64 | 12.30 | 0.66 | 11.93 | 0.40 |
|  |  |  | Nodes (count) | 1822.50 | 1739.50 | 1905.50 | 166.00 | 1817.47 | 119.58 |
|  |  |  | BD (mm^-2^) | 9.88 | 9.70 | 10.13 | 0.44 | 9.91 | 0.35 |
|  |  |  | FAZ area (mm²) | 0.30 | 0.24 | 0.38 | 0.14 | 0.32 | 0.13 |
|  |  | 0 | VAD (%) | 40.28 | 39.80 | 41.17 | 1.37 | 40.52 | 1.28 |
|  |  |  | TVL (mm) | 181.84 | 174.37 | 185.40 | 11.02 | 180.55 | 7.24 |
|  |  |  | VLD (mm/mm²) | 11.84 | 11.35 | 12.07 | 0.72 | 11.76 | 0.47 |
|  |  |  | Nodes (count) | 1760.50 | 1673.50 | 1833.50 | 160.00 | 1753.78 | 122.18 |
|  |  |  | BD (mm^-2^) | 9.68 | 9.44 | 9.88 | 0.44 | 9.70 | 0.35 |
|  |  |  | FAZ area (mm²) | 0.32 | 0.25 | 0.39 | 0.14 | 0.33 | 0.12 |
|  |  | 5 | VAD (%) | 38.48 | 38.11 | 39.26 | 1.15 | 38.70 | 1.43 |
|  |  |  | TVL (mm) | 171.72 | 168.60 | 178.12 | 9.53 | 172.81 | 6.88 |
|  |  |  | VLD (mm/mm²) | 11.18 | 10.98 | 11.59 | 0.62 | 11.25 | 0.45 |
|  |  |  | Nodes (count) | 1592.00 | 1516.25 | 1677.00 | 160.75 | 1598.94 | 118.08 |
|  |  |  | BD (mm^-2^) | 9.25 | 9.00 | 9.50 | 0.50 | 9.24 | 0.35 |
|  |  |  | FAZ area (mm²) | 0.38 | 0.26 | 0.44 | 0.19 | 0.37 | 0.15 |
|  |  | 10 | VAD (%) | 37.16 | 36.61 | 38.73 | 2.12 | 37.39 | 1.67 |
|  |  |  | TVL (mm) | 166.74 | 160.11 | 173.28 | 13.18 | 166.84 | 8.56 |
|  |  |  | VLD (mm/mm²) | 10.86 | 10.43 | 11.29 | 0.86 | 10.86 | 0.56 |
|  |  |  | Nodes (count) | 1481.00 | 1412.50 | 1583.50 | 171.00 | 1478.69 | 133.99 |
|  |  |  | BD (mm^-2^) | 8.83 | 8.66 | 9.14 | 0.47 | 8.85 | 0.40 |
|  |  |  | FAZ area (mm²) | 0.39 | 0.26 | 0.46 | 0.21 | 0.39 | 0.16 |
|  | Cirrus | -10 | VAD (%) | 39.17 | 36.51 | 41.29 | 4.78 | 38.71 | 3.82 |
|  |  |  | TVL (mm) | 162.51 | 154.60 | 174.32 | 19.72 | 163.98 | 15.06 |
|  |  |  | VLD (mm/mm²) | 10.58 | 10.07 | 11.35 | 1.28 | 10.68 | 0.98 |
|  |  |  | Nodes (count) | 1443.00 | 1337.75 | 1660.00 | 322.25 | 1497.47 | 245.22 |
|  |  |  | BD (mm^-2^) | 9.13 | 8.68 | 9.66 | 0.98 | 9.13 | 0.69 |
|  |  |  | FAZ area (mm²) | 0.65 | 0.53 | 0.80 | 0.27 | 0.63 | 0.21 |
|  |  | -5 | VAD (%) | 38.03 | 36.29 | 40.84 | 4.56 | 38.20 | 3.76 |
|  |  |  | TVL (mm) | 159.10 | 152.91 | 171.94 | 19.04 | 161.42 | 14.84 |
|  |  |  | VLD (mm/mm²) | 10.36 | 9.96 | 11.20 | 1.24 | 10.51 | 0.97 |
|  |  |  | Nodes (count) | 1405.00 | 1308.75 | 1644.25 | 335.50 | 1473.69 | 241.11 |
|  |  |  | BD (mm^-2^) | 9.05 | 8.70 | 9.77 | 1.07 | 9.10 | 0.68 |
|  |  |  | FAZ area (mm²) | 0.68 | 0.51 | 0.84 | 0.33 | 0.69 | 0.23 |
|  |  | 0 | VAD (%) | 38.00 | 36.39 | 40.75 | 4.36 | 38.03 | 3.73 |
|  |  |  | TVL (mm) | 159.52 | 153.93 | 171.87 | 17.94 | 161.11 | 14.66 |
|  |  |  | VLD (mm/mm²) | 10.39 | 10.02 | 11.19 | 1.17 | 10.49 | 0.95 |
|  |  |  | Nodes (count) | 1434.00 | 1334.00 | 1661.75 | 327.75 | 1476.13 | 243.49 |
|  |  |  | BD (mm^-2^) | 9.03 | 8.60 | 9.65 | 1.05 | 9.10 | 0.71 |
|  |  |  | FAZ area (mm²) | 0.73 | 0.55 | 0.93 | 0.38 | 0.72 | 0.26 |
|  |  | 5 | VAD (%) | 37.39 | 36.23 | 40.26 | 4.03 | 37.63 | 3.45 |
|  |  |  | TVL (mm) | 157.11 | 151.17 | 170.83 | 19.66 | 159.52 | 13.78 |
|  |  |  | VLD (mm/mm²) | 10.23 | 9.84 | 11.13 | 1.29 | 10.39 | 0.90 |
|  |  |  | Nodes (count) | 1399.50 | 1340.75 | 1669.50 | 328.75 | 1460.00 | 228.03 |
|  |  |  | BD (mm^-2^) | 9.03 | 8.66 | 9.61 | 0.95 | 9.07 | 0.67 |
|  |  |  | FAZ area (mm²) | 0.76 | 0.56 | 0.92 | 0.36 | 0.75 | 0.30 |
|  |  | 10 | VAD (%) | 36.74 | 35.20 | 40.09 | 4.89 | 37.06 | 3.59 |
|  |  |  | TVL (mm) | 152.90 | 148.69 | 168.07 | 19.38 | 156.79 | 13.95 |
|  |  |  | VLD (mm/mm²) | 9.96 | 9.68 | 10.94 | 1.27 | 10.21 | 0.91 |
|  |  |  | Nodes (count) | 1399.00 | 1292.00 | 1627.00 | 335.00 | 1439.66 | 229.00 |
|  |  |  | BD (mm^-2^) | 9.13 | 8.63 | 9.55 | 0.92 | 9.08 | 0.68 |
|  |  |  | FAZ area (mm²) | 0.91 | 0.70 | 1.08 | 0.38 | 0.89 | 0.29 |
|  | Triton | -10 | VAD (%) | 38.64 | 37.73 | 39.53 | 1.81 | 38.57 | 1.55 |
|  |  |  | TVL (mm) | 165.14 | 159.20 | 173.09 | 13.89 | 165.79 | 10.35 |
|  |  |  | VLD (mm/mm²) | 10.75 | 10.36 | 11.27 | 0.91 | 10.79 | 0.67 |
|  |  |  | Nodes (count) | 1519.00 | 1418.50 | 1615.75 | 197.25 | 1529.91 | 126.03 |
|  |  |  | BD (mm^-2^) | 9.26 | 9.07 | 9.39 | 0.32 | 9.22 | 0.30 |
|  |  |  | FAZ area (mm²) | 0.47 | 0.34 | 0.63 | 0.28 | 0.48 | 0.21 |
|  |  | -5 | VAD (%) | 38.50 | 37.30 | 39.63 | 2.33 | 38.37 | 1.65 |
|  |  |  | TVL (mm) | 166.04 | 156.03 | 173.01 | 16.98 | 164.39 | 10.59 |
|  |  |  | VLD (mm/mm²) | 10.81 | 10.16 | 11.26 | 1.10 | 10.70 | 0.69 |
|  |  |  | Nodes (count) | 1509.00 | 1398.75 | 1597.50 | 198.75 | 1502.59 | 127.04 |
|  |  |  | BD (mm^-2^) | 9.11 | 8.98 | 9.27 | 0.29 | 9.13 | 0.29 |
|  |  |  | FAZ area (mm²) | 0.50 | 0.36 | 0.61 | 0.25 | 0.49 | 0.20 |
|  |  | 0 | VAD (%) | 38.78 | 37.51 | 39.18 | 1.67 | 38.49 | 1.41 |
|  |  |  | TVL (mm) | 163.76 | 158.47 | 172.87 | 14.41 | 165.00 | 9.63 |
|  |  |  | VLD (mm/mm²) | 10.66 | 10.32 | 11.26 | 0.94 | 10.74 | 0.63 |
|  |  |  | Nodes (count) | 1529.00 | 1411.75 | 1590.00 | 178.25 | 1503.38 | 113.94 |
|  |  |  | BD (mm^-2^) | 9.13 | 8.98 | 9.24 | 0.27 | 9.10 | 0.26 |
|  |  |  | FAZ area (mm²) | 0.54 | 0.42 | 0.64 | 0.21 | 0.51 | 0.17 |
|  |  | 5 | VAD (%) | 38.45 | 37.39 | 39.27 | 1.88 | 38.51 | 1.38 |
|  |  |  | TVL (mm) | 163.13 | 158.57 | 172.05 | 13.48 | 165.27 | 9.88 |
|  |  |  | VLD (mm/mm²) | 10.62 | 10.33 | 11.20 | 0.88 | 10.76 | 0.64 |
|  |  |  | Nodes (count) | 1495.00 | 1440.75 | 1600.50 | 159.75 | 1511.16 | 114.97 |
|  |  |  | BD (mm^-2^) | 9.13 | 9.02 | 9.30 | 0.29 | 9.14 | 0.26 |
|  |  |  | FAZ area (mm²) | 0.55 | 0.46 | 0.63 | 0.17 | 0.54 | 0.16 |
|  |  | 10 | VAD (%) | 38.20 | 37.30 | 39.60 | 2.30 | 38.37 | 1.47 |
|  |  |  | TVL (mm) | 162.71 | 156.18 | 172.01 | 15.83 | 164.24 | 9.17 |
|  |  |  | VLD (mm/mm²) | 10.60 | 10.17 | 11.20 | 1.03 | 10.69 | 0.60 |
|  |  |  | Nodes (count) | 1509.50 | 1433.00 | 1602.75 | 169.75 | 1508.25 | 113.01 |
|  |  |  | BD (mm^-2^) | 9.21 | 9.04 | 9.36 | 0.31 | 9.18 | 0.30 |
|  |  |  | FAZ area (mm²) | 0.58 | 0.49 | 0.71 | 0.22 | 0.58 | 0.18 |
